# Ziv-aflibercept in Diabetic Macular Edema: Relation of Subfoveal Choroidal Thickness with Visual and Anatomical Outcomes

**DOI:** 10.18502/jovr.v18i2.13182

**Published:** 2023-04-19

**Authors:** Siamak Moradian, Masoud Soheilian, Mahsan Asadi, Abdolreza Baghi, Hamid Safi, Seyed-Hossein Abtahi

**Affiliations:** ^1^Ophthalmic Epidemiology Research Center, Research Institute for Ophthalmology and Vision Science, Shahid Beheshti University of Medical Sciences, Tehran, Iran; ^2^Ocular Tissue Engineering Research Center, Research Institute for Ophthalmology and Vision Science, Shahid Beheshti University of Medical Sciences, Tehran, Iran; ^3^Ophthalmic Research Center, Research Institute for Ophthalmology and Vision Science, Shahid Beheshti University of Medical Sciences, Tehran, Iran

**Keywords:** Center-involved Diabetic Macular Edema, Central Macular Thickness, Intravitreal, Subfoveal Choroidal Thickness, Ziv-Aflibercept

## Abstract

**Purpose:**

To evaluate the effects of intravitreal ziv-aflibercept injections (IVZ) on subfoveal choroidal thickness (SCT) as well as on central macular thickness (CMT) and on best corrected visual acuity (BCVA) changes in eyes with center-involved diabetic macular edema (CI-DME).

**Methods:**

Fifty-seven eyes of 36 patients with CI-DME were included in this prospective interventional case series. Structural optical coherence tomography (OCT) and enhanced depth imaging OCT were performed at baseline followed by three monthly 1.25 mg IVZ injections. Changes of SCT, CMT, and BCVA at each follow-up session were assessed. The association between baseline SCT and its monthly changes with final visual and anatomical outcomes were also assessed.

**Results:**

CMT at baseline, and at the first, second, and third month follow-up sessions were 396 ± 119, 344 ± 115, 305 ± 89, and 296 ± 101 μm, respectively (*P*-value < 0.001). SCT at baseline, and at months one, two, and three were 236 ± 47, 245 ± 56, 254 ± 54, and 241 ± 54 μm, respectively (*P*-value > 0.99). Corresponding figures for BCVA were 0.58 ± 0.29, 0.47 ± 0.31, 0.4 ± 0.24, and 0.37 ± 0.23 LogMAR, respectively (*P*-value < 0.001). There was a statistically significant positive correlation between BCVA and CMT changes following IVZ injections (*P*-value < 0.001). However, there were no significant correlations between SCT changes and visual acuity (VA) and CMT changes following IVZ injections.

**Conclusion:**

IVZ improved visual outcomes and macular thickness profiles in patients with CI-DME. However, IVZ had no significant effect on SCT. Baseline SCT and its monthly changes had no association with visual and anatomical outcomes.

##  INTRODUCTION

Diabetic macular edema (DME) is the main cause of vision loss in diabetic patients.^[1,2]^. The use of anti-vascular endothelial growth factors (anti-VEGFs) is currently the main therapeutic choice for DME.^[3]^ Ziv-Aflibercept (Zaltrap, Sanofi-Aventis US, LLC, Bridgewater, New Jersey, USA and Regeneron Pharmaceuticals, Inc, Tarrytown, New York, USA) is an FDA-approved drug for treatment of colorectal and metastatic cancers. As an analogue of aflibercept, ziv-aflibercept is a monoclonal antibody that binds to VEGF A and B of various isoforms and placental growth factor, which also possesses a good safety profile and is of lower cost.^[4,5]^ There is a growing body of evidence that intravitreal ziv-aflibercept (IVZ) improves visual outcomes and macular thickness profiles in patients with DME.^[6,7,8,9,10,11]^ However, the changes of choroidal thickness in eyes with DME treated with IVZ and the effects of subfoveal choroidal thickness (SCT) on visual and anatomical outcomes remain unknown.

Subfoveal choroidal thickness measurement performed by enhanced depth optical coherence tomography (EDI-OCT) in DME cases treated with anti-VEGFs has been proposed as a predictor of therapeutic outcomes.^[12]^ However, the correlation between baseline SCT and treatment responses in patients with DME remains controversial.^[3,13,14]^ Thinning of SCT might aggravate retinal hypoxia and accentuate VEGF secretion resulting in increased breakdown of the blood retinal barrier (BRB) and consequent DME deterioration.^[15,16]^ Therefore, the association of SCT with visual and anatomical outcomes should be elucidated.

In this study, we investigated the effects of IVZ on SCT as well as on central macular thickness (CMT) and on best corrected visual acuity (BCVA) changes in eyes with center-involved diabetic macular edema (CI-DME). A possible correlation between SCT changes and visual and anatomical outcomes of DME cases treated with IVZ is questioned.

##  METHODS

In this prospective interventional case series, cases of CI-DME were enrolled for intravitreal pharmacotherapy at Labbafinejad Medical Center, Tehran, Iran between April 2019 and April 2020.

The study protocol was reviewed and approved by the Ethics committee, Ophthalmic Research Center, Shahid Beheshti University of Medical Sciences, Tehran, Iran, and the approval number was IR.SBMU.ORC.REC.1394.05. In addition, the protocol of the study complied with the guidelines for human studies and the tenets of Declaration of Helsinki. Informed consent was obtained from all participants.

Based on the standard protocols, all patients underwent three monthly IVZ injections (1.25 mg/0.05 ml).^[17]^ Complete ocular examinations, Spectralis EDI-OCT (Heidelberg Engineering, Heidelberg, Germany), and SD OCT were performed at baseline and repeated one month after each IVZ injection. All visits and imaging procedures took place between 9 am and 12 am. BCVA was evaluated using the Snellen chart. CMT was assessed based on previously described OCT software methodology (the 1-mm Early Treatment Diabetic Retinopathy Study (ETDRS) circle centered on the fovea).^[18]^ SCT was measured manually as the distance between the RPE hyperreflective line and the chorio-scleral junction. Only patients with CMTs of > 250 µm and BCVA readings between 20/40 and 20/320 were included. Patients with proliferative diabetic retinopathy (PDR), concomitant macular disease, a history of intravitreal injections of anti-VEGFs or corticosteroids or retinal photocoagulation within three months of enrollment, a history of pars plana vitrectomy, refractive errors higher than ± 5 diopters, a history of cardiovascular accidents, glaucoma, pregnancy, and uncontrolled systemic hypertension were excluded.

### Statistical Analysis

Data were presented using mean, standard deviation, median and range, frequency, and percentage. Paired *T*-test was used to assess the improvement within the groups and *T*-test was used to evaluate the difference among the groups.

Minimal sample size needed for all variables was calculated as ≥ 45 individuals (G*Power software [latest ver. 3.1.9.7; Heinrich-Heine-Universität Düsseldorf, Düsseldorf, Germany]) to achieve a certain power (effect size dz = 0.5; α error probability = 0.05; power [1-β error probability] = 0.95). We also used generalized estimating equations (GEE) to consider the possible correlation of the results in the eyes. Correlation of variables was assessed using Pearson's correlation coefficient. All statistical analyses were performed using the SPSS (IBM Corp. Released 2017. IBM SPSS Statistics for Windows, Version 25.0. Armonk, NY: IBM Corp.). *P*-value < 0.05 was considered statistically significant.

**Table 1:** Baseline characteristics and demographic features.


	***N*** ** (%) **
Eye	OD	30 (52.6%)
	OS	27 (47.4%)
Lens status	Pseudophakic	20 (35.1%)
	Phakic	37 (64.9%)
History of MPC	No	31 (54.4%)
	Yes	26 (45.6%)
History of injection	No	28 (49.1%)
	Yes	29 (50.9%)
Naïve	No	36 (63.2%)
	Yes	21 (36.8%)
Age	Mean ± SD	63.3 ± 5.93
Number of injection	Mean ± SD	3.93 ± 1.03
Duration of diabetes (yr)	Mean ± SD	12.37 ± 4.23
	
	
MPC, macular photocoagulation; OD, right eye; OS, left eye; SD, standard deviation		

**Table 2:** Best-corrected visual acuity, central macular thickness, subfoveal choroidal thickness before and at one, two, and three months after monthly injection of intravitreal ziv-aflibercept.


	**Baseline**	<@orange**Month 1**			<@orange**Month 2**			<@orange**Month 3**		
	**Values**	**Values**	**Changes**	***P*** **-value**	**Values**	**Changes**	***P*** **-value**	**Values**	**Changes**	***P*** **-value**
BCVA	0.58 ± 0.29	0.47 ± 0.31	–0.15 ± 0.22	0.202	0.4 ± 0.24	–0.19 ± 0.26	0.002	0.37 ± 0.23	–0.22 ± 0.27	< 0.001
CMT	396 ± 119	344 ± 115	–52 ± 123	0.057	305 ± 89	–91 ± 132	< 0.001	296 ± 101	–102 ± 144	< 0.001
SFCT	236 ± 47	245 ± 56	9 ± 55	> 0.99	254 ± 54	18 ± 55	0.197	241 ± 54	7 ± 61	> 0.99
	
	
*P*-value calculated using paired *T*-test BCVA, best-corrected visual acuity; CMT, central macular thickness; SFCT, subfoveal choroidal thickness										

**Table 3:** Correlation between central macular thickness changes, best corrected visual acuity changes, and subfoveal choroidal thickness changes at one, two, and three months after monthly injection of intravitreal ziv-aflibercept.


**Time**		**CMT and BCVA**	**SFCT and CMT**	**SFCT and BCVA**
Month 1	Pearson Correlation	0.436**	0.186	–0.025
	*P*-value	0.002	0.165	0.864
Month 2	Pearson Correlation	0.446**	0.006	0.028
	*P*-value	0.001	0.966	0.845
Month 3	Pearson Correlation	0.456**	0.104	0.047
	*P*-value	0.001	0.453	0.743
	
	
**Correlation is significant at the 0.01 level (2-tailed) BCVA, best-corrected visual acuity; CMT, central macular thickness; SFCT, subfoveal choroidal thickness				

##  RESULTS

Fifty-seven eyes of 36 patients with CI-DME were included in this prospective interventional case series. During the study period, no patient missed follow-up sessions or required further treatments (e.g., laser photocoagulation, vitrectomy) and no complications related to IVZ were recorded in patients (signs of intraocular inflammation, change in the lens status, and systemic complications). Past medical history and ocular examination findings were summarized in Table 1.

CMT decreased progressively through IVZ monthly injections. A marginally significant decrease was recorded at the one month follow-up (*P* = 0.057); however, compared to the baseline a significant decrease was recorded after the second and third months. Progressive BCVA improvement was also observed after monthly IVZ injections. Significant changes were recorded after the second and third months as compared to the baseline. No significant changes in SCT were observed after monthly IVZ injections as compared to baseline [Table 2].

At each follow-up interval, the level of improvement of BCVA was correlated with the amount of CMT reduction. However, SCT did not correlate with changes in BCVA and CMT at follow-up visits [Table 3].

##  DISCUSSION 

Ziv-aflibercept (Zaltrap) is a monoclonal antibody approved for the treatment of metastatic colorectal cancer. Like its analogue, aflibercept, Ziv-aflibercept blocks all isoforms of VEGF A, B, and placental growth factor (PlGF). While Ziv-aflibercept (Zaltrap) has higher osmolarity than aflibercept, studies showed no difference between the two drugs in the safety profile. Malick et al compared the toxicity of aflibercept, Ziv-aflibercept, bevacizumab, and ranibizumab on RPE cells in media culture. They observed mild mitochondrial toxicity following bevacizumab and ziv-aflibercept injections.^[19]^ In an experimental study, 0.05 ml IVZ had no deleterious effect on the osmolarity of the vitreous.^[20]^ Mansour et al showed the effect of IVZ in patients with DME without any intraocular toxicity.^[9]^ Chhablani et al demonstrated the effect of IVZ injection on patients with neovascular AMD with no intraocular toxicity or retinal electrophysiological abnormalities.^[21]^ Additionally, no significant adverse effects related to IVZ were detected, at least in a short-term follow-up. However, long-term adverse effects of IVZ remains unknown.

Variable results have been reported regarding choroidal thickness changes in patients with DME.^[14,15,22]^ There are reports of reduced choroidal thickness in these patients, which may be the consequence of choroidal vascular obstruction and development of non-perfusion areas similar to retinal vascular alterations.^[15,22]^ A histopathological study revealed thinning and attenuation of the choriocapillaris layer with subsequent reduction of choroidal thickness in diabetic retinopathy.^[14]^ Meanwhile, some studies showed increase of choroidal thickness in patients with DME. VEGF overexpression causes dilation and congestion of choroidal vessels that result in thicker choroid in patients with DME. Therefore, anti-VEGFs for DME treatment could theoretically reduce the permeability and thickness of choroidal layers. However, no significant association was found between anti-VEGF therapy and choroidal thickness changes in patients with CI-DME.

The effect of anti-VEGF treatment on choroidal thickness in DME has already been evaluated.^[3,12,13,14,23,24,25]^ Several studies reported reduction of choroidal thickness following intravitreal injection of bevacizumab (IVB).^[3,12,23]^ Rayess et al revealed that thicker baseline SCT was associated with better anatomic and functional responses. They suggested baseline SCT as a predictor of DME treatment response to IVB.^[12]^ Yiu et al reported no correlation between SCT reduction and changes in visual acuity (VA) and macular thickness.^[3]^ In a clinical trial, no significant changes were observed in choroidal thickness following IVB injection in DME.^[24]^ Recently, it has been shown that intravitreal aflibercept had more effect on SCT in DME as compared to intravitreal ranibizumab.^[25]^

Despite previous investigations using various forms of anti-VEGFs, the effect of IVZ on choroidal thickness in DME has not been evaluated so far. In our study, SCT did not significantly change after IVZ injection in patients with CI-DME. In addition, despite adequate sample size and statistical power of the analysis, we observed no correlation between SCT and changes of CMT and BCVA after IVZ injections. It might be related to different receptor density and sensitivity of retinal and choroidal vasculature to VEGF. It is believed that choroidal thickness reduction in DME may be related to factors other than VEGF. Hence, VEGF may not be the sole contributor to choroidal thickness changes in DR.^[26]^

There were few limitations in the current study. It lacked a contralateral eye comparison. The absence of correlation between IVZ and changes in choroidal thickness might be related to the short follow-up time of the study. In addition, we did not compare the effect of IVZ with other forms of anti-VEGFs. SCT changes might not represent the changes of the entire choroidal tissue. A more detailed assessment of the choroidal vascular index and stromal area could be a better way to assess the choroidal structure changes in response to intravitreal anti-VEGF injections.

In summary, IVZ is a promising form of anti-VEGF drug used to improve visual and anatomical outcomes in patients with CI-DME without significant short-term toxicity. The visual and anatomical effects of IVZ on DME are not correlated with any changes in SCT.

##  Financial Support and Sponsorship

None.

##  Conflicts of Interest

None of the authors have any conflicts of interest to declare.
